# Perinatal hypoxia and the risk of severe Molar-Incisor Hypomineralisation (MIH): a retrospective analysis of the pH value of umbilical arterial blood after birth

**DOI:** 10.1007/s40368-021-00622-5

**Published:** 2021-04-12

**Authors:** C. Hoberg, C. Klein, D. Klein, C. Meller

**Affiliations:** 1 Paediatric Dental Practice, Kinder + Zahnarzt Berlin, Berlin, Germany; 2 Department of Conservative Dentistry, Periodontology, Endodontology and Paediatric Dentistry, Centre of Dentistry, Oral Medicine, and Maxillofacial Surgery, University Hospital and Faculty of Medicine, Tübingen, Germany; 3 Paediatric Dental Practice, Meller Schlauzahn, Waiblingen, Germany

**Keywords:** Molar-Incisor Hypomineralisation (MIH), Umbilical cord blood, PH value, Perinatal hypoxia, Cesarean section

## Abstract

**Purpose:**

Molar-Incisor Hypomineralisation (MIH) remains a widespread developmental disorder of the teeth with a still largely unknown etiology. Perinatal events were blamed in previous studies for the development of MIH.

The aim of the present study was to evaluate the influence of perinatal hypoxia—determined by the pH value of the umbilical cord blood—and to investigate its correlation with severe MIH retrospectively. In addition, cesarean section was recorded as differentiation variable.

**Methods:**

A total number of 138 children (mean age 8.0 years ± 1.7), who were treated for severe MIH in a dental office in Berlin between the years 2008 and 2019, were included in the study. The control group was comprised of patients with the same date of birth (44 children, mean age 7.7 years ± 1.7). Information on the pH value of the arterial blood from the umbilical cord taken immediately after birth, whose recording is mandatory in Germany, was received from the parents by letter survey requesting the entries from the German Child Health Booklet.

**Results:**

In the group of the male children born without cesarean section, the pH value of the control group was significantly lower (7.19 ± 0.09) than the pH value of the MIH group (7.27 ± 0.07, *p* = 0.0008). In female children born with or without cesarean section as well as in male children born by cesarean section there were no significant differences between the MIH and control group.

**Conclusions:**

No significant association between MIH and the pH value of the umbilical cord blood or birth by cesarean section could be found in the examined patient population.

## Introduction

The Molar-Incisor Hypomineralisation (MIH) represents a health problem for the patients concerned, particularly in childhood, and describes a structural developmental anomaly of the dental enamel with systemic cause, which normally and primarily affects one or more permanent (6 years) molars and/or permanent incisors (Weerheijm et al. 2001; Lygidakis 2010). Especially in its severe manifestation, the so-called “severe MIH”, the affected teeth show increased risk of developing painful hypersensitivity, deep carious lesions and/or chewing-induced tooth wear/fractures immediately after tooth eruption (Lygidakis et al. 2010; Weerheijm et al. 2001; Fagrell 2011). The increased tendency of the affected teeth to develop pulpitis often complicates treatment by local anesthesia not being effective. Consequently, children with MIH also show significantly more dental treatment phobia than unaffected children (Jalevik and Klingberg 2002). Therefore, due to complications caused by MIH at a young age, the treatment of children can also often only be carried out under general anesthesia/sedation, which in turn often represents an additional psychological and financial burden for the affected children and their parents.

According to systematic reviews, the global occurrence of MIH is estimated to be highly prevalent by affecting on average about 13–15% (Jalevik 2010; Zhao et al. 2018; Dave and Taylor 2018; Schwendicke et al. 2018) of the population beginning at the age of 8 years. However, differences among regions or countries are strong and can vary from about 2–40% (Jalevik 2010). Authors point out, however, that the results are to be regarded with reservation, since the criteria for the evaluation were different and, in some regions, only few people had been examined. It is estimated that MIH currently affects 878 million people, with 17.5 million of new cases every year. Of these figures, an additional proportion of over 27% was determined to have MIH requiring therapeutic treatment, which corresponds to around 240 million existing cases and 5 million new cases per year worldwide (Schwendicke et al. 2018). These figures make MIH a major global health problem.

Despite intensive research, the etiology of MIH remains largely unknown, and thus the chances for better prevention of the disease are slight (Alaluusua 2010; Silva et al. 2016; Fatturi et al. 2019). Since there is no sufficient evidence to validate the causative factors, the treatment of affected teeth has so far been limited mainly to symptom-related, caries prophylactic and restorative measures. Unfortunately, this concept of waiting for MIH lesions to develop is not in accordance with the goal of preventing a disease before it manifests clinically. The potential causes that are being discussed are among others early childhood diseases, vitamin deficiency, infant antibiotic administration and perinatal complications (Alaluusua 2010; Silva et al. 2016; Fatturi et al. 2019). Multifactorial and genetic components are also being considered (Vieira and Kup 2016). As far as specific events that take place during the process of birth are concerned, some studies described a statistically significant correlation with cesarean section (Lygidakis et al. 2008; Pitiphat et al. 2014; Garot et al. 2016) and perinatal hypoxia (Garot et al. 2016). The latest systematic review and meta‐analysis on possible etiologic factors associated with MIH concludes also that situations which could be linked to possible events of perinatal hypoxia (*i.e.,* “*cesarean delivery, delivery complications and respiratory diseases*”) are significantly associated with MIH. Nevertheless, since this evidence was obtained “*from studies with serious limitations, such as the risk of bias, imprecision and inconsistencies*”, these conclusions should be carefully interpreted (Fatturi et al. 2019). The parameters for determining perinatal hypoxia were a weakness in previous MIH studies, as occurrence of hypoxia was not verified by blood test, but rather by subjective parameters as expression of perinatal hypoxia. Therefore, studies are needed that seek more objective methods that can help identify or corroborate risk factors related to MIH development (Alaluusua 2010; Silva et al. 2016; Fatturi et al. 2019). Over the past four decades, numerous studies established pH tests of umbilical cord blood at birth as an objective and practicable screening measure to discard and/or evaluate stages of hypoxia (fetus as being acidotic), which has led to this procedure becoming a standard procedure for neonatal evaluation (Yeh et al. 2012; Malin et al. 2010; ACOG Committee on Obstetric Practice 2006; Thorp and Rushing 1999; Chen and Liu 2013; Victory et al. 2004).

Due to the problems described above, the aim of the present study was to investigate the influence of perinatal hypoxia, determined by the pH value of arterial blood extracted from the umbilical cord immediately after birth, and its correlation with the development of severe MIH. The null hypothesis is that there is no correlation between the pH value of umbilical cord blood used to measure perinatal hypoxia and the occurrence of severe MIH.

## Materials and methods

### Selection of subjects

In the present study, children were selected from the patient pool of a pediatric dental practice with two locations in Berlin. 584 cases with severe MIH met the inclusion criteria in the treatment group (MIH group) and were contacted. Of this number, a total of 138 children [mean age (8.0 ± 1.7) years] treated in the dental practice between 2008 and 2019 participated in this study with the consent of their parents, who provided additional data (i.e., record of the pH value of the arterial blood from the ‘Child Health Booklet’). The selection of these cases was based on the fact that permanent first molars with severe MIH were always restored with preformed metal crowns in the above-mentioned pediatric dental practice. The corresponding item for billing allowed the identification of the restored cases with the help of the dental practice management software used in the dental office (Z1, Version 2.71, Compudent AG, Germany). Only cases in which an MIH diagnosis had been recorded in the patient’s file prior to the restoration procedure with the metal crown were considered, in accordance with EAPD criteria (Weerheijm et al. 2003).

For each child willing to participate from the MIH group, usually two children without MIH but with the same date of birth were selected from the patient pool of the same dental practice as control group. Altogether 272 cases without MIH were contacted. The parents of 44 children [mean age (7.7 ± 1.7) years] responded and provided additional data for the control group.

The exclusion criteria considered all dental pathologies not specifically associated with severe MIH. Only data received with the consent of the parents of the participating children were included. If subjects or their parents were not willing to participate, they were excluded from this study.

The methodology of the present study was based on retrospective data analysis from patient records and did not include any type of direct clinical trials with human participants by any of the authors. All data derived from the medical records of the patients were handled in accordance with the principles of the Declaration of Helsinki updated in 2013 and in compliance with the local ethical and legal regulations in force (Berufsordnung der Zahnärztekammer Berlin).

### Data determination

The determination of the perinatal hypoxia at birth was based on the pH value of the arterial blood from the umbilical cord that was collected and recorded immediately after birth. In Germany, this value is routinely determined and recorded in the ‘Child Health Booklet’, as part of the first preventive care examination (the so-called “U1” examination) of the newborn immediately after birth (Lawrenz 2020).

### Data collection and analysis

In the present study, the parents of the children were informed by letter about the intention of the study and asked both about the pH value of the umbilical cord blood, as documented in the Child Health Booklet, and whether a cesarean section was performed during delivery.

Statistical analyses were performed with the program JMP 14.2 (SAS Institute Inc., Cary, USA). First, the data (mean ± standard deviation) were tested for a normal distribution using the Shapiro–Wilk W test with *p* > 0.05. Statistically significant differences were performed for numerical variables (gender and pH value) using the *t* test and for qualitative variables (gender and birth) using the Chi-square test at a significance level of *α* = 0.05.

## Results

The response rate in the MIH group was 23.3% and in the control group 16.2%.

The demographic distribution of the study groups by age, gender and birth is shown in Table 1. There is no statistically significant difference between the MIH group and the control group in any of the three variables. The proportion of cesarean sections in the MIH group is 25.7%, and in the control group 22.7%.

**Table 1:** Demographic distribution of study groups (mean ± standard deviation)

	MIH (*n* = 136)	Control (*n* = 44)	*p* value
Age^a^	7.99 ± 1.66	7.66 ± 1.73	0.26
Gender *n* (%)^b^			
Male	65 (47.8%)	22 (50%)	0.80
Female	71 (52.2%)	22 (50%)	
Birth *n* (%)^b^			
Cesarean section	35 (25.7%)	10 (22.7%)	0.68
Normal delivery	101 (74.2%)	34 (77.2%)	

^a^*t* test

^b^Chi^2^-test

**Fig. 1:**
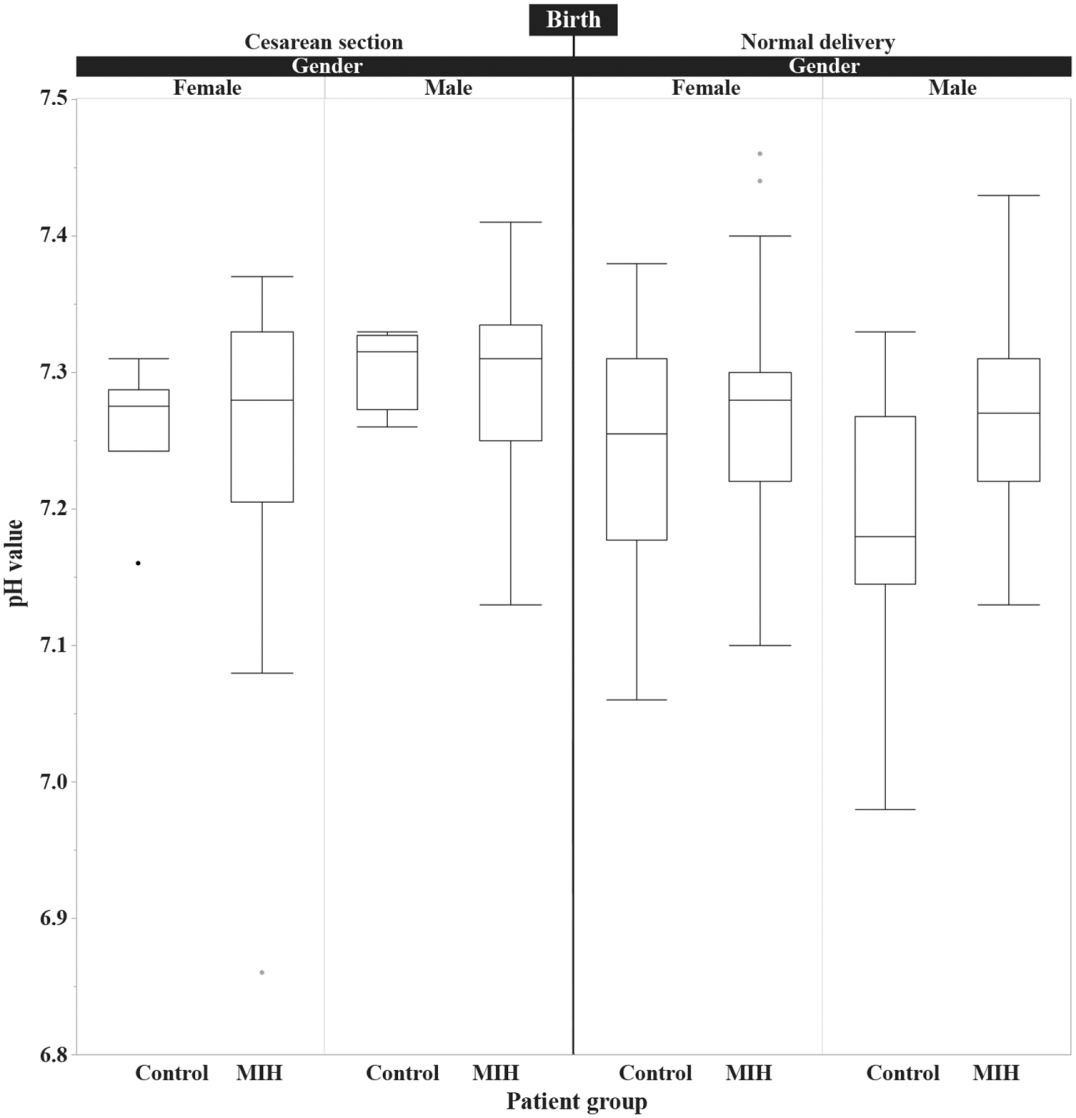
Box plot of the pH values of the umbilical cord blood differentiated by gender and type of birth

The pH values of the umbilical cord blood are depicted in Table 2. There were no significant differences between the MIH and control group for the female children with and without cesarean section and for the male children with cesarean section. Concerning male children without cesarean section, the pH value was significantly lower with 7.19 ± 0.09 in the control group than the pH value of the MIH group with 7.27 ± 0.07 (*p* = 0.0008). The difference of the pH mean values was 0.08. The pH value distribution for the groups is shown in Fig. 1.

**Table 2:** pH value of the umbilical cord blood differentiated by gender and birth (mean ± standard deviation)

Birth	Gender	MIH	Control	*p* value
Cesarean section				
	Male	7.29 ± 0.08 (*n* = 18)	7.31 ± 0.03 (*n* = 4)	0.72
	Female	7.24 ± 0.12 (*n* = 17)	7.26 ± 0.05 (*n* = 6)	0.66
	Total	7.27 ± 0.10 (*n* = 35)	7.28 ± 0.05 (*n* = 10)	0.67
Normal delivery				
	Male	7.27 ± 0.07 (*n* = 47)	7.19 ± 0.09 (*n* = 18)	0.0008*
	Female	7.24 ± 0.10 (*n* = 54)	7.27 ± 0.08 (*n* = 16)	0.13
	Total	7.27 ± 0.07 (*n* = 101)	7.21 ± 0.10 (*n* = 34)	0.001*
Total (birth and gender)		7.27 ± 0.08 (*n* = 136)	7.23 ± 0.09 (*n* = 44)	0.004*

*t* test

## Discussion

To clarify possible individual etiological factors responsible for the development of MIH is useful, especially the unique events immediately around birth, as this is part of the phase in which most MIH lesions on teeth develop. The present study used the pH of the umbilical cord blood to determine hypoxia during birth. Severe fluorotic changes are not endemic in the region under study and severe cases of MIH were retrospectively defined by steel crown restorations of permanent molars in childhood. Thus, both variables could be validly determined in retrospect without confusing them with fluorosis and/or milder forms of MIH. Furthermore, it was assumed that in case of severe MIH, a possible proportional correlation to etiological factors is more likely.

The assessment of perinatal hypoxia has been a weak point in previous MIH studies, as up to now no objective parameter has been used to confirm that the newborn has indeed suffered an incident of hypoxia during delivery. In a recent study, Garot et al. (2016) addressed this issue and used, therefore, data from the subjects’ medical records at birth. However, even in this study, hypoxia was not measured by one parameter measured directly in the blood, but by a variety of parameters that express perinatal hypoxia. Because of the difficulties in finding a suitable hypoxia risk model in MIH related studies and the difficult calculation of the influence of single risk factors, the present study suggest to use ‘*hypoxia activity*’ (determined by the pH value of the umbilical cord) instead of subjective ‘*hypoxia risk*’ factors (like for instance preterm birth, long labor events, the mode of delivery, Apgar score, etc.), when a correlation to MIH development is investigated. Since hypoxia activity signifies the impact of the sum of several risk factors and their interdependencies acting together on the blood, it could objectively be used to clarify whether this plays a real role in the etiology of MIH.

Since the work of James et al. (1958) reported that umbilical cord blood pH analysis can be used to reflect fetal hypoxia during birth, the test has become the gold standard assessment procedure for neonatologists to determine acid–base status at birth. Nowadays, the standard procedure is to draw arterial blood from the clamped umbilical cord immediately after birth. The pH difference between arterial and venous blood is usually small and, therefore, not relevant (Ghi et al. 2018). This also reduces the significance of errors in blood collection (vein/artery) and in the documentation of the pH value. Multiple studies demonstrated that this analysis can help to objectively confirm and identify babies suffering from asphyxia during birth. The test provides important information to confirm perinatal adverse events and the neonatal status (Yeh et al. 2012; Malin et al. 2010; ACOG Committee on Obstetric Practice 2006; Thorp and Rushing 1999; Chen and Liu 2013; Victory et al. 2004). Therefore, it is nowadays widely accepted that the analysis of the blood pH immediately after birth should be added for newborns (as an additional test to even the Apgar score) to increase the objectivity in the identification of relevant oxygenation problems suffered during delivery. Apgar scores have been associated with a high rate of neonatal asphyxia false positives (Chen and Liu 2013).

No connection could be observed between perinatal hypoxia or cesarean section and the incidence of severe MIH in the examined patient population. This differs from previous studies in which hypoxia (Garot et al. 2016) and cesarean section (Garot et al. 2016; Lygidakis et al. 2008; Pitiphat et al. 2014) were associated with MIH. However, the present study is the first to explore a possible hypoxia state during delivery by analysing the pH value of the umbilical cord blood. In the study of Garot et al. (2016), hypoxia was not measured directly by a blood test, but indirectly presumed by parameters that could be related to perinatal hypoxia.

In accordance with this study, Brogardh-Roth et al. (2011) also found no association between cesarean birth and the presence of MIH in a study of preterm babies. In the patient population of the present study, the proportion of cesarean births in patients with MIH (25.7%) is only slightly higher than in the control group (22.7%) and only slightly lower compared to the proportion of births after cesarean section performed in Berlin (27.3%) in 2010 (GBE-Bund 2010).

A worldwide, significant general decrease in cases of peripartum asphyxia, as well as a normally fast recovery of children after hypoxic phases during birth, has been observed in the past decades (Yeh et al. 2012; Malin et al. 2010). The normal pH value of the arterial blood collected from the umbilical cord has been found to be 7.24 ± 0.07 (Victory et al. 2004). Minimal variations (values less than 1 or 2 standard deviations below the mean) from these values are associated with a substantial increase in the risk of requiring admission to neonatal intensive care units or the need for assisted ventilation (Victory et al., 2004). If the blood pH falls below the normal range, the condition is referred to as “acidosis”. An extensive study investigating the relationship of umbilical cord pH and serious neonatal side-effects originated from a hospital in Oxford and investigated over 50,000 patients over a time period of 18 years. In that study, the mean arterial pH value was 7.22, with a threshold for significantly increased neurological problems at a pH value below 7.10. The “ideal” pH value indicated in that study with the least neurological damage was in the range between pH 7.26–7.30 (Yeh et al. 2012). As limiting factor, it should be noted that in England (in comparison to the present study) the umbilical cord pH value is only recorded if newborn asphyxia is suspected and not as a routine procedure as it is in Germany. In the examined patient data, the mean of the pH value determined by the umbilical cord blood is in the ideal range according to Yeh et al. (2012) as indicated above. A statistically significant difference between MIH and control group in terms of pH is surprisingly found in the group of male patients without cesarean section, a result that rather contradicts the idea of a connection between hypoxia during delivery and MIH. Thus, perinatal hypoxia as the cause for MIH does not seem likely in this investigation. The significant difference in the group of male patients with normal delivery cannot be explained. However, the pH value of 7.19 is just at the threshold of mild acidosis and thus not clinically relevant. The significant differences between the summaries from the “normal delivery” and the “birth and gender” group can be attributed to the effect in the group of male patients with normal delivery. Here, the pH values are also not in the range of acidosis and the difference is also not clinically relevant.

Even though this study did not establish a connection to severe MIH, the question remains, whether a connection to milder forms of MIH exists after all. The study of Garot et al. (2016), which points out that hypoxia during birth or cesarean section could represent a risk factor for the development of MIH, did not analyse what role these factors could play in the occurrence of cases showing severe MIH. Since oxygenation issues and cesarean section procedures during delivery are very well handled medically nowadays, it is more likely that the majority of severe MIH cases we see in children today are unrelated to incidents in connection with either hypoxia or cesarean section. Thus, with the observed pH values, the present study possibly merely proves the high quality standards in medicine that nowadays prevail at birth and not a connection between either perinatal hypoxia or cesarean section with MIH. Thus, hypoxia during birth does not seem to cause damage to the ameloblast function, similar to birth by cesarean section, which is actually a less strenuous process for the child.

In summary, where the examined data is concerned, it seems that there were hardly any fluctuations in the normal pH of the blood during birth. Nevertheless, these children developed severe MIH. Herewith, a connection between hypoxia and resulting MIH cannot be completely excluded, but the majority of MIH incidents in Germany are likely to be found in children without caesarean delivery or oxygen deficiency during birth. The small control group, in comparison to the MIH group, represent a limitation in the study. Preferably, future studies should include children with MIH from different geographical areas. Further studies are required to gain more knowledge about the origin of MIH.

## Conclusions

Within the limitations of the present study the following conclusions can be made:

- No significant correlation between the pH value of the umbilical cord arterial blood and the occurrence of severe MIH could be found in the examined patient population.
- There was no significant correlation between the occurrence of severe MIH and cesarean section.
- Merely a statistically significant but not clinically relevant difference between severe MIH and the control group in terms of pH value was found in male children without cesarean section.
